# Unpacking Multimodal Verb Input in English: Insights From Typically Developing Infants and Those Who are Deaf and Hard of Hearing

**DOI:** 10.1111/infa.70127

**Published:** 2026-09-20

**Authors:** Feile Meng, Isobel Horsfall‐Turner, Cassim Hussain, Iris Nomikou, Rana Abu‐Zhaya

**Affiliations:** ^1^ Division of Psychology and Language Sciences University College London London UK; ^2^ School of Psychology, Sport, and Health Sciences University of Portsmouth Portsmouth UK

**Keywords:** action‐naming, deaf and hard‐of‐hearing, mother‐infant interaction, multimodal communication, verb input

## Abstract

This study offers a descriptive account of multimodal verb input to Deaf and Hard of Hearing (DHH) and Typically Developing (TD) infants across crucial points in verb acquisition. Using the North American English Ambrose (2016) corpus, we analysed mother‐infant play interactions at 13 and 22 months, and explored caregivers' verb input, its combination with caregiver action and in response to infant action. Across development, mothers increased the frequency and diversity of their verb input while retaining high levels of multimodal scaffolding such that verbs were tightly coordinated with actions. Multimodal verb input varied as a function of age and agent. When producing verbs in response to infant actions, mothers of younger infants used verbs that labeled the actions that infants were performing, whereas when infants were older, mothers more often used verbs that were related to aspects of the actions performed but did not label them. In verb input that accompanied their own actions, mothers produced more actions that were related to the meaning of the verbs more frequently than actions that depicted the meaning of the verbs, and this was more common in the input to older infants. Crucially, while verb input to DHH infants was less lexically diverse, it was more frequently multimodally packaged suggesting that mothers of DHH infants scaffold verb input by reducing its complexity and increasing its referentiality with multimodal cues that can facilitate the construction of verb meaning.

## 1 Introduction

Caregiver‐infant interactions are rich social contexts through which infants and caregivers co‐construct meaning (Tamis‐LeMonda et al. 2014; Tomasello and Carpenter 2007). This view is consistent with embodied theories of language, which propose that infants learn language both by observing others' interactions and by actively participating in them, with meaning grounded in perceptual and motor processes shaped by sensorimotor experiences (Barsalou 1999; Glenberg 1997; Wellsby and Pexman 2014). Complementing this perspective, social‐pragmatic theories highlight the importance of the social environment in guiding language learning (Carpenter et al. 1998). Building on these accounts, studies revealed that within everyday social interactions, infants' actions often elicit multimodal responses from caregivers through labeling, touch, or gestures that align closely with the infant's focus of attention and facilitate word learning (Nomikou et al. 2017; Suarez‐Rivera et al. 2022; Tamis‐Lemonda et al. 2013). Importantly, this process is dynamic and bidirectional: infants' behaviors shape the input they receive from caregivers, while caregiver responses in turn scaffold learning, reflecting the reciprocal nature of early language development (Chen et al. 2021; Tamis‐LeMonda 2023).

These early language environments are characterized by their multimodality; caregiver speech is accompanied by actions, gestures, and touch (Kosie and Lew‐Williams 2024), and the synchrony of these cues with speech renders it more accessible to infants (Abu‐Zhaya et al. 2017; Gogate et al. 2000; Nomikou and Rohlfing 2011; Nomikou et al. 2017; Rodrigo et al. 2020) and helps them decipher word meaning (Custode and Tamis‐LeMonda 2020; Nomikou et al. 2017; Yu and Smith 2012). For instance, infants show more frequent gaze shifts from mother to object and better word‐mapping when their caregivers synchronize spoken words with object motion (Gogate et al. 2006). Additionally, caregiver touch that is temporally aligned with and congruent to speech, aids in speech segmentation and word learning (Seidl et al. 2024; Tincoff et al. 2018). This combination of cues reduces referential ambiguity allowing infants to better associate words with their meanings (Baldwin et al. 1996; Gogate et al. 2006).

Yet, much of this evidence is limited to studies on noun learning, whereas relatively very little research has examined how multimodal cues scaffold verb learning. This scarcity might be linked to the view, which is perpetuated due to the structure of commonly studied languages (e.g., English), that verbs are more challenging to learn than nouns (Golinkoff and Hirsh‐Pasek 2008). Yet, work on indigenous languages like Tseltal shows that because some verbs may incorporate properties of their objects, acquiring their meaning does not require the same degree of abstraction as languages that do not have this property (Brown 1998). In English, verbs typically depict actions, processes, or states, which are inherently dynamic and dependent on context (Golinkoff and Hirsh‐Pasek 2008). Verbs often require children to pay attention to the timing and sequence of actions, which can be emphasized through synchronized gestures or actions (Vigliocco et al. 2019). These unique features make verbs a particularly interesting component of multimodal communication. Compared to nouns, verbs offer more opportunities for using various multimodal cues, some of which might uniquely accompany verbs. For example, when a caregiver produces the verb *jump*, they can use gestures, body movements, and object manipulation to mimic the action, providing cues that help toddlers associate verbs with events (Golinkoff and Hirsh‐Pasek 2008; Nomikou et al. 2017).

Recent work examined the multimodality of verbs in the input to typically developing (TD) infants. At 6 months, mothers synchronize verbs with actions that depict the verbs' meanings, such that verbs either match caregivers' actions or label infants' actions (Nomikou et al. 2017). Later in development, between 13 and 18 months, West et al. (2022) showed that mothers are more likely to provide a verb label in response to an infant action: mothers' whole‐body verbs co‐occur with infants' whole‐body actions and mothers' manual verbs co‐occur with infants' manual actions. However, Rodrigo et al. (2020) showed that between 12 and 36 months, mothers' verbs continue to either match their own actions or the child's and the proportion of mothers' verbs matching mother versus child actions remains stable between 12 and 36 months. Finally, Zhang et al. (2025) showed that at 12 months, mothers utilize distinct verbal and non‐verbal patterns for different types of verbs; specifically, the syntactic contexts in which verbs appear in mothers' utterances and their embodied characteristics (e.g., gaze, manual actions) depend on the type of verb (e.g., action/mental verbs). Together, these findings confirm that infants' activities serve as “catalysts” for caregiver input, accentuating that verb acquisition can be embedded in reciprocal, action‐oriented exchanges between caregivers and infants.

Despite these advances, we have yet to fully characterize developmental changes in multimodal verb input, particularly because children's verb learning and caregiver input undergo marked change during the second year of life. By 13.5 months, infants can segment verbs from fluent speech (Nazzi et al. 2005) and show evidence of understanding some verbs in experimental studies (Frewin 2023; Nomikou et al. 2019). At this stage, caregiver speech is reported to be grounded in the here‐and‐now (Tamis‐Lemonda et al. 2013) even when it varies by activity (Tamis‐LeMonda et al. 2019). Across the second year, toddlers' expressive verb vocabularies expand rapidly in size and diversity (Fenson et al. 2006; Longobardi et al. 2016), and by around 21–22 months, children can draw on more abstract linguistic information, such as transitive syntax, to establish verb meaning even in the absence of a corresponding event (Arunachalam et al. 2013). In parallel, caregiver input becomes more linguistically complex (Huttenlocher et al. 2010; Rowe 2012) and verb input in particular increases in frequency and diversity between 13 and 18 months (West et al. 2022). Beyond linguistic form, caregivers also adapt the multimodal packaging of input to children's developmental level (Gogate et al. 2000; Ko et al. 2023; Tamis‐Lemonda et al. 2013); for example, they temporally synchronize target words with object motion and touch more frequently in the input to preverbal infants than toddlers (Gogate et al. 2000). Yet, it should be noted that existing research on the development of multimodal input is very scarce, and existing studies either include very small samples, focus on specific multimodal combinations, or are theoretical models with little underpinning data. Together, these factors indicate that the period between infants' first and second birthday is well suited for capturing how multimodal verb input is dynamically structured and that detailed studies on the development of this input are indispensable.

One documented aspect of such structure is that verbs can label either caregivers' or infants' actions (Nomikou et al. 2017; Rodrigo et al. 2020; West et al. 2022); however, it is unclear whether caregivers deploy these strategies differently between infants' first and second birthdays given infants' lexical development and previously documented changes in caregivers' use of multimodal cues. Studies suggest that caregivers make the input perceivable and tangible for their infants through temporal synchrony and iconicity/depiction, particularly early in development (Nomikou and Rohlfing 2011; Nomikou et al. 2017) when a close alignment between word and world might be especially supportive for establishing referentiality (Perniss and Vigliocco 2014). Yet, the degree of redundancy of information from different sensory modalities, that is whether they reinforce or supplement each other, might vary with age and context. For example, speech and gesture can work together to convey the same semantic information, thereby reinforcing the speech signal, but could also present different information to disambiguate the speech signal (Iverson et al. 1999). Such multimodal combinations are often in response to infants' ongoing actions, like their gaze toward the mother, creating a special form of multimodal synchrony, that is both temporally and interactionally contingent (Rohlfing and Nomikou 2014). On a social‐interactive level, such responsive input creates a pragmatic frame in which language is co‐constructed through shared activities (Rohlfing et al. 2016); on a sensory level, it provides time‐locked experiences that link linguistic forms to embodied events (Tamis‐LeMonda et al. 2014). For instance, in the input to 6‐month‐olds (Nomikou et al. 2017) and 12‐ to 36‐month‐olds (Rodrigo et al. 2020), multimodally reinforced verbs are produced in response to infants' bodily actions. These insights indicate that multimodal verb input can be temporally tied to caregivers' and infants' actions with possible shifts throughout development.

Yet, evidence from previous work indicates that *some aspects* of multimodal input vary not only with infants' age (Gogate et al. 2000; Ko et al. 2023; Tamis‐Lemonda et al. 2013), but also their sensory and communicative capabilities (Abu‐Zhaya et al. 2019; Chen et al. 2019; Goldin‐Meadow and Saltzman 2000; Kadlaskar et al. 2020; Koester and Lahti‐Harper 2010; Talbott et al. 2015). Of specific interest in the current study are infants who are deaf or hard of hearing (DHH); these infants have varying degrees of auditory access, ranging from mild‐to‐severe hearing loss (hard of hearing) to profound hearing loss (deaf) (World Health Organization 2025), and some of them benefit from using hearing aids (HAs) or cochlear implants (CIs). Although CIs and HAs can support access to spoken language (to varying degrees), the auditory input available to DHH infants can be limited or less detailed than that available to hearing infants (Lederberg et al. 2013). For DHH infants, auditory access to spoken language may be reduced or variable across everyday contexts, such that linguistic information is not consistently perceived and taken up. Over time, these moment‐to‐moment “misses” can accumulate, constraining children's cumulative language experience and contributing to variability in language outcomes (Moeller and Tomblin 2015). In response, caregivers may adapt their communicative strategies to accommodate the needs of their DHH child. Indeed, studies show that some mothers of DHH children produce shorter and simpler utterances, more directives and reduced lexical diversity than mothers of hearing peers (Bergeson et al. 2006; Fagan et al. 2014; Vanormelingen et al. 2016), while some maintain the same quantity of talk (Ambrose et al. 2015; Chen et al. 2019). Such adaptations are also evident in the use of non‐speech cues. Differences in sensory access between DHH infants and their typically hearing caregivers create a sensory mismatch that may lead caregivers to adapt their language input to support shared attention and communicative alignment through more accessible visual and tactile cues (Abu‐Zhaya et al. 2019; Chen et al. 2019; Goldin‐Meadow and Saltzman 2000; Koester and Lahti‐Harper 2010). In sum, these studies offer important groundwork for investigating how multimodal language can be fine‐tuned and matched to the child's needs and sensory skills.

The current study offers a unique contribution to this niche in language acquisition research: using a longitudinal corpus of mother‐infant play interactions with TD and DHH English‐learning infants (Ambrose 2016), we provide a thorough descriptive account of multimodal verb input in English, and how it varies by development and hearing status. By focusing on two crucial points in lexical development, that is, 13 and 22 months, and comparing TD to DHH infants, we examine how multimodal verb input in English can be used to support linguistic development and scaffold sensory deprivation. Specifically, we address the following questions:

1. What does the multimodal packaging of verbs look like and how does it change as infants develop?

We expect that as infants grow, caregivers will produce more verbs, both in terms of frequency and diversity, but that the multimodal packaging of these verbs will become less prominent. Furthermore, we expect that verb input provided to infants that matches the actions of the infant will be more prevalent than input that matches the actions of the mother, and that infant‐led multimodal verbs will be more common in interactions with older infants. Finally, we expect that earlier in development, multimodal verb input will be significantly more depictive of the meaning of verbs than later.

2. Does multimodal verb packaging differ depending on the infant's hearing status?

We expect mothers of DHH infants will integrate actions with verbs more frequently than mothers of TD infants. Additionally, we expect that in the DHH sample, most multimodal input will be matched to mothers' actions rather than infants' actions or co‐constructed by both. Finally, we expect that caregivers of DHH infants will reinforce verb input by using more depictive actions than actions that are only related to the verb.

## 2 Methods

### 2.1 Corpus Description

Participants were drawn from a longitudinal North American English corpus (Ambrose 2016). The corpus includes data from 40 mother‐child dyads: 22 dyads include DHH infants, who failed their newborn hearing screening and/or were diagnosed with bilateral hearing loss by 6 months, and 18 dyads include TD infants, matched by chronological age who passed a hearing screening. DHH infants differed in their auditory profiles, including variation in the onset of use of hearing technologies (HAs and CIs) and in the degree of hearing loss in the better ear, as documented in the corpus records. All children were being raised in English speaking homes, and none had cognitive delays or neurological diagnoses. Maternal education was matched between the groups but was not included in the corpus documentation.

Dyads were video recorded in a laboratory playroom for 30 minutes during each of five visits at different time points in development. Mothers were instructed to engage in typical play with their infants using age‐appropriate toys, while children moved freely around the room. The interactions were transcribed orthographically. All video recordings and transcripts are accessible from the CHILDES database (MacWhinney 1995).

### 2.2 Current Study Sample

The current study focused on interactions recorded when children were 13 and 22 months old. Dyads were included if they provided usable video data at both time points and if the analysed segments captured interactions exclusively between mother and child. Of the original 40 dyads, 15 were excluded. Six DHH dyads were excluded due to missing video data at one or both time points. An additional 6 dyads (4 DHH, 2 TD) were excluded because the analysed segments included sustained involvement of third parties (e.g., siblings, fathers, or experimenters). To obtain more comparable group sizes, 3 TD dyads were randomly excluded.

The final sample included 25 dyads (TD group: *n* = 13, 6 males, 7 females; DHH group: *n* = 12, 4 males, 8 females) with two videos from each for a total of 50 videos. At the first data collection point, ages ranged from 13.26 to 13.67 months (*M* = 13.51, SD = 0.12) for the TD group and 13.20–14.97 months (*M* = 13.86, SD = 0.55) for the DHH group. At the second point, ages ranged from 22.33 to 23.00 months (*M* = 22.62, SD = 0.18) for the TD group and 21.37–23.17 months (*M* = 22.55, SD = 0.50) for the DHH group. All children in the DHH group were fitted with HAs before the first visit. By the first visit, four children in the DHH group had received CIs, another five received their first CI between 13 and 22 months, and three did not utilize CIs.

### 2.3 Data Coding

Using ELAN (Version 6.7; Wittenburg et al. 2006), and in line with previous work (Depowski et al. 2015; Kondaurova et al. 2013), we coded and analysed a 6‐min segment from each video, from minute 12:00 to 18:00. By choosing a video segment from the middle, we aimed to avoid the initial phase where dyads are just getting used to the new environment, and the final part where tiredness might influence their behaviors. Although the original corpus includes transcription of sign language at the first timepoint, sign use was not transcribed at the second timepoint and was not included as a separate analytic category in the present study. Accordingly, in the current analyses, verb tokens are only spoken, and manual behaviors (actions and gestures) are coded without distinguishing conventionalized signs from gestures.

#### 2.3.1 ELAN File Setup

For each video, we created an ELAN file that included the transcript and a coding template with six tiers: *verb*, *verb_infinitive*, *comments*, *labeling*_*episode*, caregiver action (*cg_action*), and infant‐led action (*inf_led*). The template allowed for unified coding across the dataset, and the transcripts facilitated finding verb labels in maternal speech.

#### 2.3.2 Verb Coding

To find the verbs in mothers' utterances, we searched for “v” in the morphology tier imported from the transcript (*%mor@MOT*) and manually identified verbs that were not marked as verbs in the transcript by examining the content of mothers' utterances. All verbs were included, irrespective of their tense. Auxiliary verbs and their conjugations were not coded. Annotations for each verb were created in the *verb* tier. The infinitive forms of these verbs were annotated on the *verb_infinitive* tier; discrepancies with the transcript were noted on the *comments* tier.

#### 2.3.3 Action Coding

After coding the verbs, on the *labeling_episode* tier, we marked the 3 s before the onset and the 3 s after the offset of each verb (Bornstein et al. 2008; Custode and Tamis‐LeMonda 2020; West et al. 2022), labeled “pre” and “post,” respectively, and together marked the labeling episode.

Next, coders watched the labeling episode surrounding each verb and separately coded the caregivers' and infants' actions. Caregiver's actions surrounding each verb were coded using the *cg_action* tier and were classified into: Caregiver Depictive (CGD), Caregiver Relevant (CGR), and Not Codable (NC). CGD actions were those in which the caregiver depicted the meaning of the verb, for example, saying “we can *stir* some eggs” while stirring. CGR refers to any verb‐relevant actions performed by the caregiver that did not directly depict the meaning of the verb. These actions could involve indirect engagement such as pointing or manipulating an object related to the verb without executing the verb's meaning, for example saying “do you want to *look* in here?” while opening the refrigerator door. Actions that were not codable due to visibility issues were classified as NC.

On the *inf_led* tier, we coded infant actions where the action trajectory began within the 3 s preceding the verb label. Infant actions that occurred after the verb were not coded as these could have been responses to the input. The coding categories used were: Infant Depictive (ID), Infant Relevant (IR), and NC. ID actions captured instances where the infant's action was matched by the caregiver's verb; for example, if the infant was dancing and the mother labeled this action as “*dance*”. IR actions included instances where the infant performed an action, and the caregiver labeled it with a verb that was relevant but not directly descriptive of the action. For example, when the infant picked up a telephone receiver, the mother said, “are you *talking* to Nana?”.

Coding caregiver and infant actions in this manner (see Supporting Information S1 for further details) allowed us to provide a comprehensive description of multimodal verb input; based on this coding scheme, verbs could be multimodal because they are preceded/accompanied/followed by a caregiver action (CGD, CGR), because they are preceded by an infant action (ID, IR), or because they are surrounded by both a caregiver and infant action (i.e., co‐constructed). Each of these options yields a “multimodal verb”.

### 2.4 Data Extraction and Further Coding

Data were exported from ELAN in a tab‐delimited file that included all codes (verbs, caregiver actions, infant actions) and their time stamps. The raw data was then inspected using a custom script which examined each verb and caregivers' and infants' actions based on their timestamps and classified each verb as being multimodal or not, along with the exact nature of multimodality (per the definition above). Upon extracting the data, we identified 189 different verb types (e.g., laugh, jump). Two coders coded whether each verb was a mental verb or an action verb. Verbs were coded as action verbs if they were related to physical motion or transfer. These could be object‐action verbs or movement verbs with observable referents, as well as verbs causing change in the environment. In contrast, mental verbs were verbs with no observable referents that related to mental states or to the senses, including psychological attitude verbs. This coding scheme was developed by combining definitions from several previous studies (Davis and Landau 2021; Laakso and Smith 2007; West et al. 2022; Zhang et al. 2025) and aimed at expanding our knowledge regarding the types of verbs in the input. In the coding process, coders consulted the supplementary materials in Zhang et al. (2025) but did not consult the videos to determine verb category. Inter‐rater reliability was conducted on the full list of 189 verbs; Cohen's kappa (Cohen 1968) was 0.61 (SE = 0.07, 95% CI [0.48, 0.74]).

## 3 Results

All analyses were conducted in R studio 2025.05.1+513 for windows. We fit linear mixed effect models for all analyses using lme4 (Bates et al. 2015) and we report unstandardized effect sizes for all significant effects (per recommendation set forth by the APA task force and in Baguley 2009; Pek and Flora 2018); where possible, we also report the 95% CI for those effect sizes.

### 3.1 Mothers' Use of Verbs: Frequency, Type, and Unique Verbs

**FIGURE 1:**
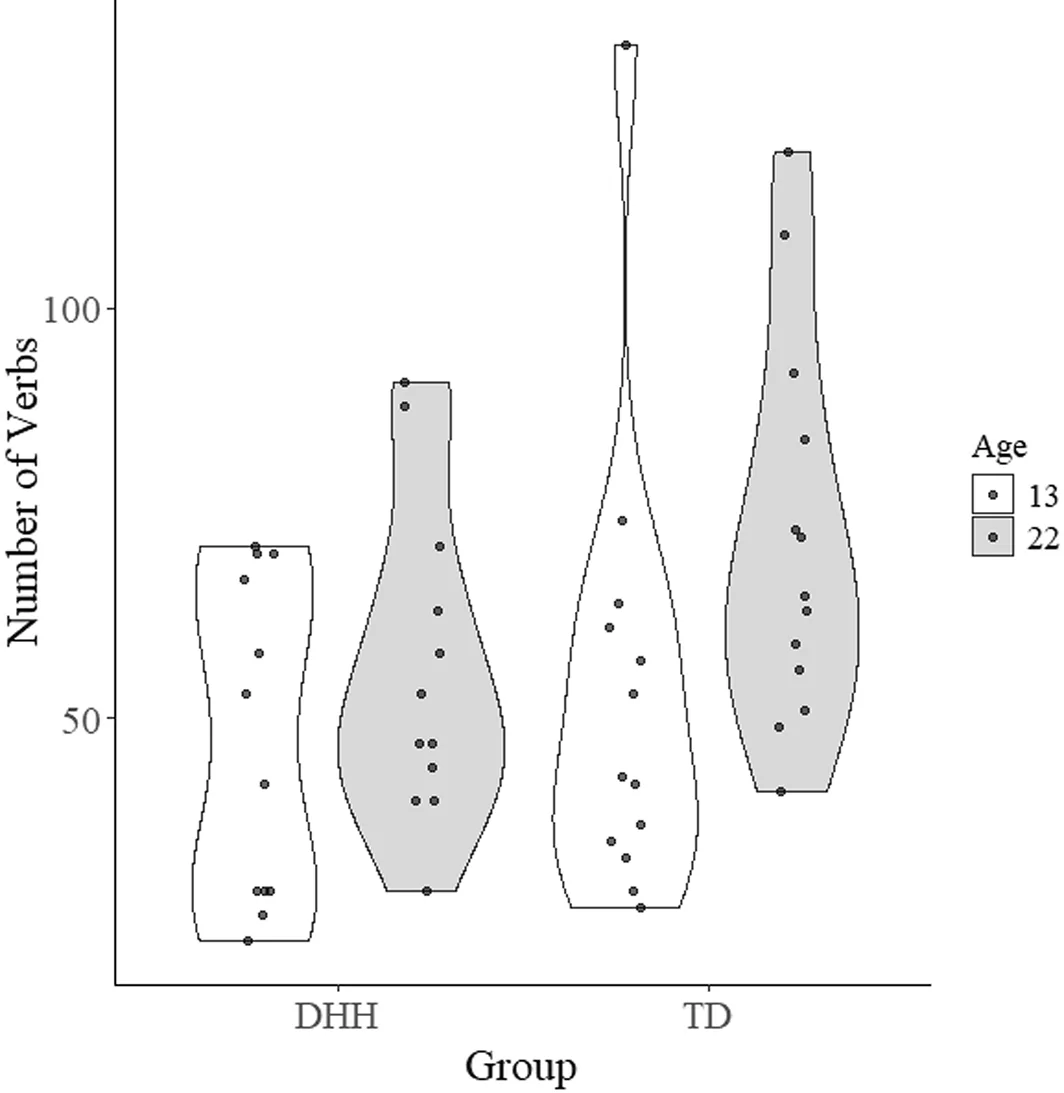
Developmental change in maternal verb frequency in the input to DHH and TD infants at 13 and 22 months.

First, we examined verb frequency in the input. With number of verbs as our dependent variable, we fitted a general linear mixed effects model with Group (DHH, TD), Age (13, 22), and their interaction as fixed effects, and by‐subject random intercepts (syntax: no_verbs ∼ Group*Age + (1|Subject)). In line with previous work on the frequency of verbs in the input to English‐learning children (West and Iverson 2017; West et al. 2023), we found a significant age effect (*β* = 13.8, SE = 5.708, *t* = 2.417, *p* = 0.024; effect size estimate: 13.8, 95% CI [1.99, 25.6]): on average, mothers produced 50.16 verbs in the input to 13‐month‐olds (range = 23–132; SD = 23.92, 95% CI [40.28, 60.03]), and 64.16 verbs in the input to 22‐month‐olds (range = 29–119, SD = 22.60; 95% CI [54.83, 73.48]). We found no effect of group (*β* = 10.724, SE = 7.2, *t* = 1.50, *p* = 0.15) or group*age interaction (*β* = 10.256, SE = 11.416, *t* = 0.898, *p* = 0.378) on the number of verbs (Figure 1).

Further, we examined the proportion of action and mental verbs out of the total number of verbs produced; we fitted a linear mixed effects model with Group (DHH, TD), Age (13, 22), Verb type (AC, ME) and their interaction as fixed effects, and by‐subject random intercepts (syntax: Proportion ∼ Group Age*VerbType + (1|Subject)). Results revealed a significant effect of verb type (*β* = −0.18, SE = 0.03, *t* = −5.94, *p* < 0.001; effect size estimate: 0.18, 95% CI [0.12, 0.24]) such that action verbs constituted a higher proportion of mothers' verb input (AC: *M* = 0.59, 95% CI [0.54, 0.63]; ME: *M* = 0.41, 95% CI [0.36, 0.45]). All other main effects, and their interactions were not statistically significant.

**FIGURE 2:**
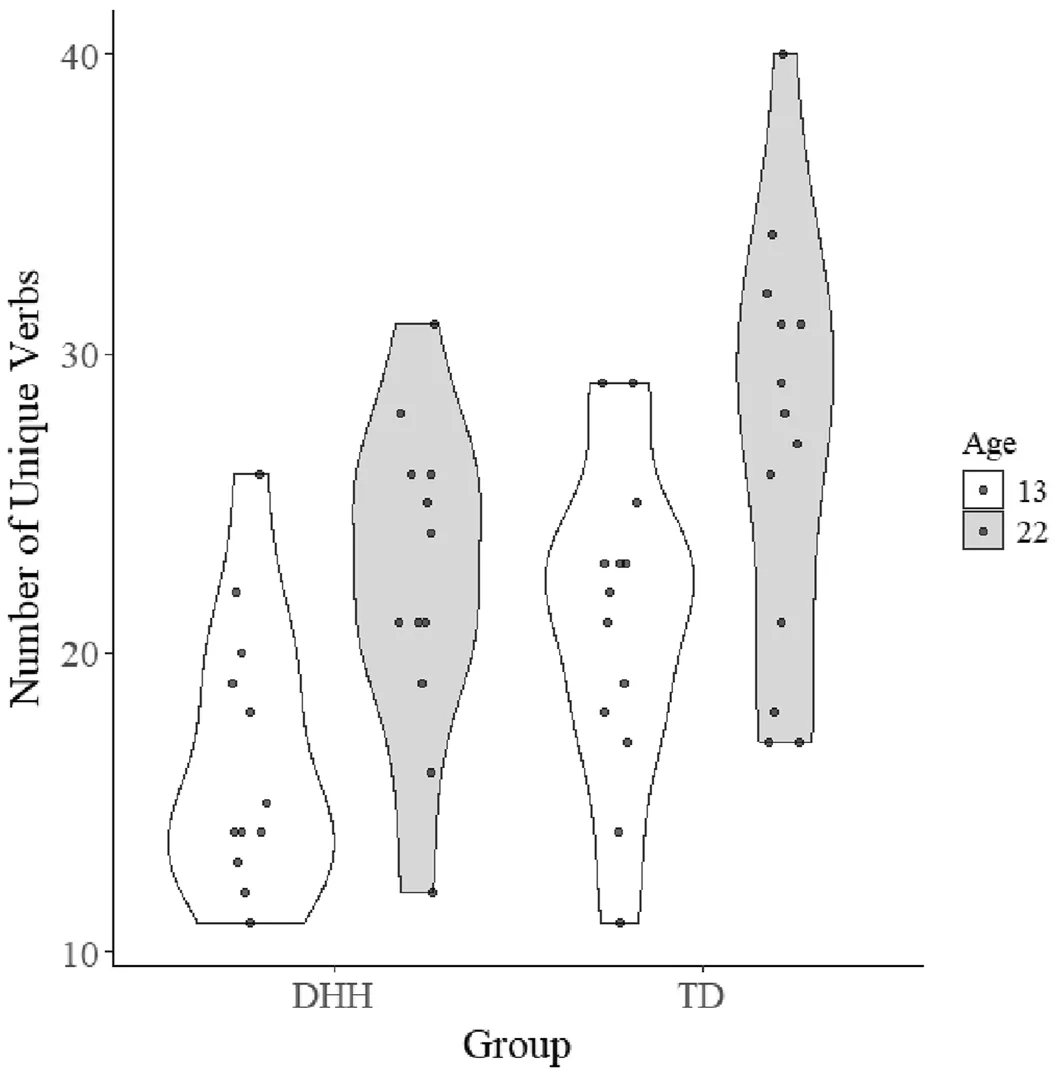
Developmental change and group differences in the number of unique verbs produced in the input to DHH and TD infants at 13 and 22 months.

Next, we examined the number of unique verbs in the input. With number of unique verbs as our dependent variable, we fitted a general linear mixed effects model with Group (DHH, TD), Age (13, 22), and their interaction as fixed effects, and by‐subject random intercepts (syntax: unique_verbs ∼ Group*Age + (1|Subject)). Results revealed a significant age effect (*β* = 5.96, SE = 1.6, *t* = 3.73, *p* < 0.001; effect size estimate: 6, 95% CI [1.23, 10.8]), such that mothers produced a larger number of unique verbs in the input to their 22‐month‐olds (*M* = 24.84, range = 12–40, SD = 6.55, 95% CI [22.13, 27.54]) compared to that produced when these infants were 13 months old (*M* = 18.88, range = 11–29, SD = 5.36, 95% CI [16.66, 21.09]). Our results also revealed a significant group effect (*β* = 4.53, SE = 1.6, *t* = 2.84, *p* < 0.01; effect size estimate: 5.92, 95% CI [1.34, 10.5]), such that mothers of TD infants produced a larger number of unique verbs (*M* = 24.05, range = 11–40, SD = 6.81, 95% CI [21.28, 26.79]) compared to mothers of DHH infants (*M* = 19.50, range = 11–31, SD = 5.70, 95% CI [17.09, 21.91]). We found no evidence for a statistically significant group*age interaction effect (*β* = −0.07, SE = 3.19, *t* = −0.024, *p* = 0.98); see Figure 2.

### 3.2 Multimodal Verb Input

**FIGURE 3:**
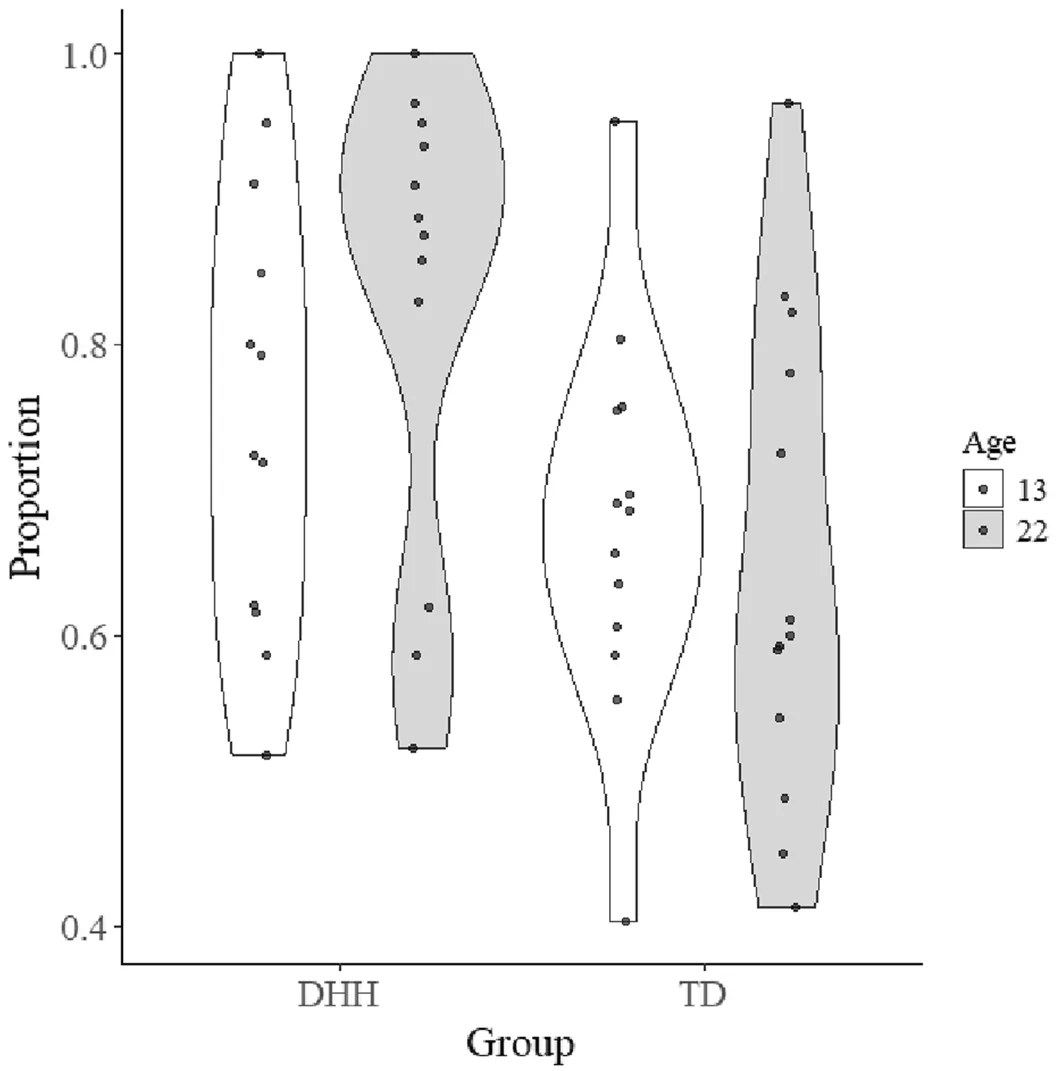
Group differences in the proportion of maternal verbs produced with multimodal cues in the input to DHH and TD infants at 13 and 22 months.

In this section, we examine the multimodal features of verbs. Multimodal verbs are verbs that were accompanied by a caregiver action (coded as either CGD or CGR), infant action (coded as either ID, IR), or both (co‐constructed). We calculated the proportion of multimodal verbs as the number of multimodally reinforced verbs divided by the total number of verbs produced by each mother at each time in development. With proportion of multimodal verbs as our dependent variable, we fitted a linear mixed effects model with Group (DHH, TD), Age (13, 22), and their interaction as fixed effects, and by‐subject random intercepts (syntax: ProportionMultimodalVerbs ∼ Group*Age + (1|Subject)). Results revealed a significant effect of group (*β* = −0.13, SE = 0.05, *t* = −2.59, *p* = 0.016; effect size estimate: 0.131, 95% CI [0.02, 0.23]) such that mothers of DHH infants produced a higher proportion of multimodal verbs (*M* = 0.79, range = 0.52–1.00, SD = 0.16, 95% CI [0.72, 0.86]) compared to mothers of TD infants (*M* = 0.66, range = 0.40–0.97, SD = 0.15, 95% CI [0.60, 0.72]). We found no effect of age (*β* = 0.02, SE = 0.03, *t* = 0.61, *p* = 0.547) or group*age interaction (*β* = −0.10, SE = 0.07, *t* = −1.43, *p* = 0.166) on the proportion of multimodal verbs that mothers produced (Figure 3).

### 3.3 Agent and Type of Multimodal Verb Input—Analysis Plan

Each multimodal verb was classified by the agent(s) of the action that rendered the verb multimodal: accompanied by a caregiver action, preceded by an infant action, or co‐constructed. Co‐constructed verbs were produced in response to an infant action and supplemented with a caregiver action and were therefore constructed by the caregiver and the infant within the same labeling episode. Caregiver and infant actions were classified by *type*: CGD, CGR, ID, IR (see Action Coding section in the methods). A verb may have been surrounded by two types of caregiver actions (CGD and CGR) or preceded by two types of infant actions (ID and IR); in those cases, the verb was classified as “mix”.

Given group differences in the proportion of multimodal verbs out of the total number of verbs in the input, in subsequent analyses, we fit separate models for each group. This approach allows us to unpack the unique patterns within each group without losing statistical power to the small sample and 3‐way interaction. For these next analyses, we calculated the proportion of verbs accompanied by a caregiver action, preceded by an infant action, or co‐constructed; these were calculated as the number of multimodally reinforced verbs of each agent type separately (caregiver, infant, co‐constructed) divided by the total number of multimodal verbs produced by each mother at each timepoint.

We then inspected multimodal verbs in more detail by examining the type of actions that accompanied them, whether they were produced by the caregiver (CGD, CGR, or mix) or the infant (ID, IR; there was no record of a mix category here). The proportions of multimodal verbs that were accompanied by either a CGD, CGR, or a mix of both were calculated as the number of CGD, CGR, or both divided by the total number of verbs that were accompanied by a caregiver action, and similarly the proportions of verbs that were accompanied by an ID or IR were calculated as the number of ID or IR verbs divided by the total number of verbs that were preceded by an infant action.

### 3.4 Multimodal Verb Labeling in the Input to DHH Infants

**FIGURE 4:**
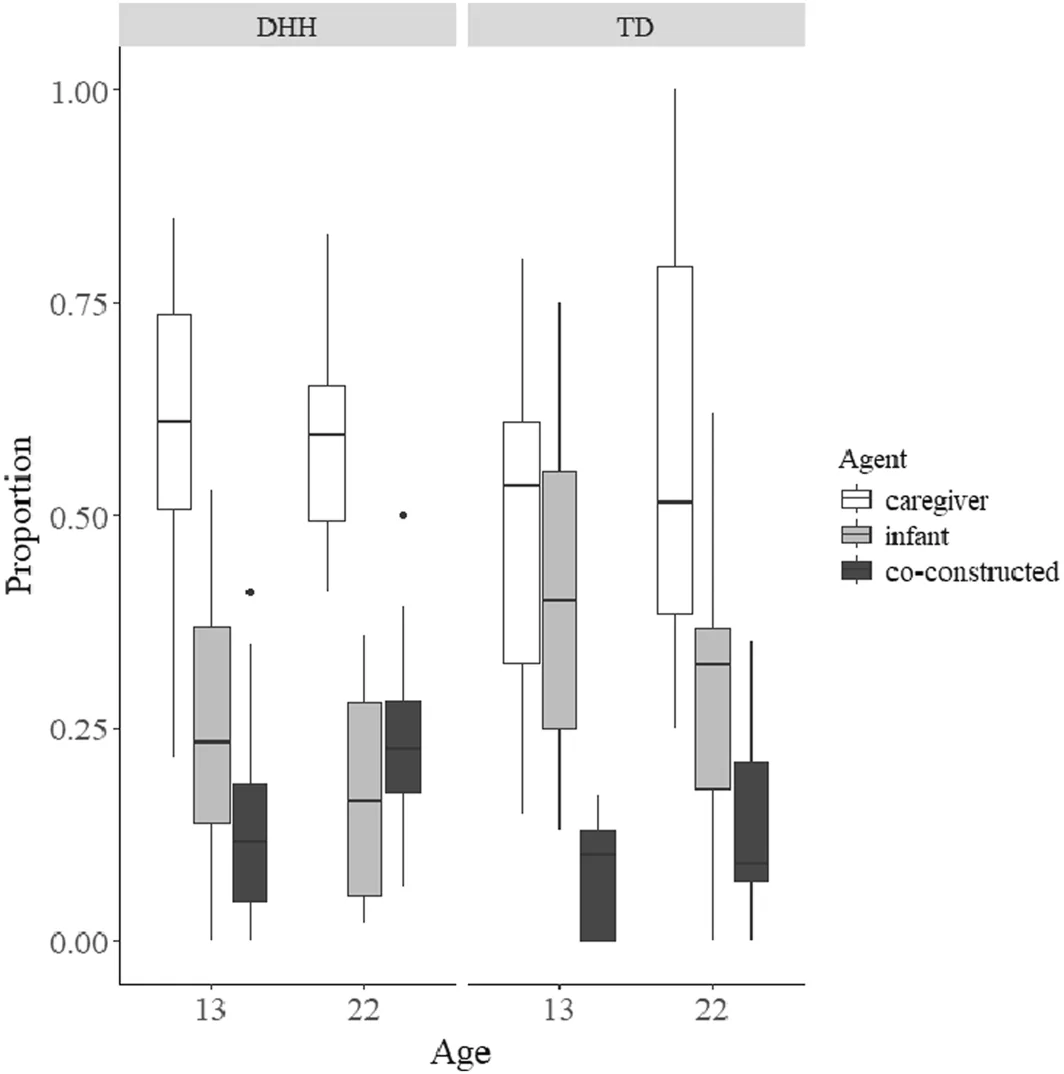
Proportion of multimodal verbs in each agent category (caregiver, infant, co‐constructed) at 13 and 22 months in the input to DHH and TD infants.

With proportion of multimodal verbs per agent type as our dependent variable, we fitted a linear mixed effects model with Age (13, 22), Agent (caregiver, infant, co‐constructed; reference level was caregiver), and their interaction as fixed effects, and by‐subject random intercepts (syntax: Proportion ∼ Age*Agent + (1|Subject)). Results revealed no significant effect of age (*β* = −0.03, SE = 0.06, *t* = −0.45, *p* = 0.657), or of any of the interactions between agent and age (age*infant: *β* = −0.04, SE = 0.08, *t* = −0.54, *p* = 0.593; age*co‐constructed: *β* = 0.12, SE = 0.08, *t* = 1.48, *p* = 0.143) on the proportion measure. However, we found a statistically significant effect of agent, such that the proportion of verbs produced with caregiver action differed from those preceded by an infant action (*β* = −0.39, SE = 0.04, *t* = −9.26, *p* < 0.001; effect size estimate: −0.38, 95% CI [−0.48, −0.28]), and those that were co‐constructed (*β* = −0.40, SE = 0.04, *t* = −9.68, *p* < 0.001; effect size estimate: −0.4, 95% CI [−0.5, −0.3]); specifically, the proportion of verbs produced with caregiver action averaged 0.60 (range = 0.22–0.85, SD = 0.15, 95% CI [0.53, 0.66]), whereas those preceded by infant action, and verbs that were co‐constructed averaged 0.21 and 0.19 respectively (infant: range = 0.00–0.53, SD = 0.15, 95% CI [0.15, 0.27]; co‐constructed: range = 0.00–0.50, SD = 0.13, 95% CI [0.14, 0.25]; Figure 4). Thus, in the input to DHH infants, verbs were more often accompanied by caregiver action rather than following infants' action or co‐constructed by the caregiver and infant.

**FIGURE 5:**
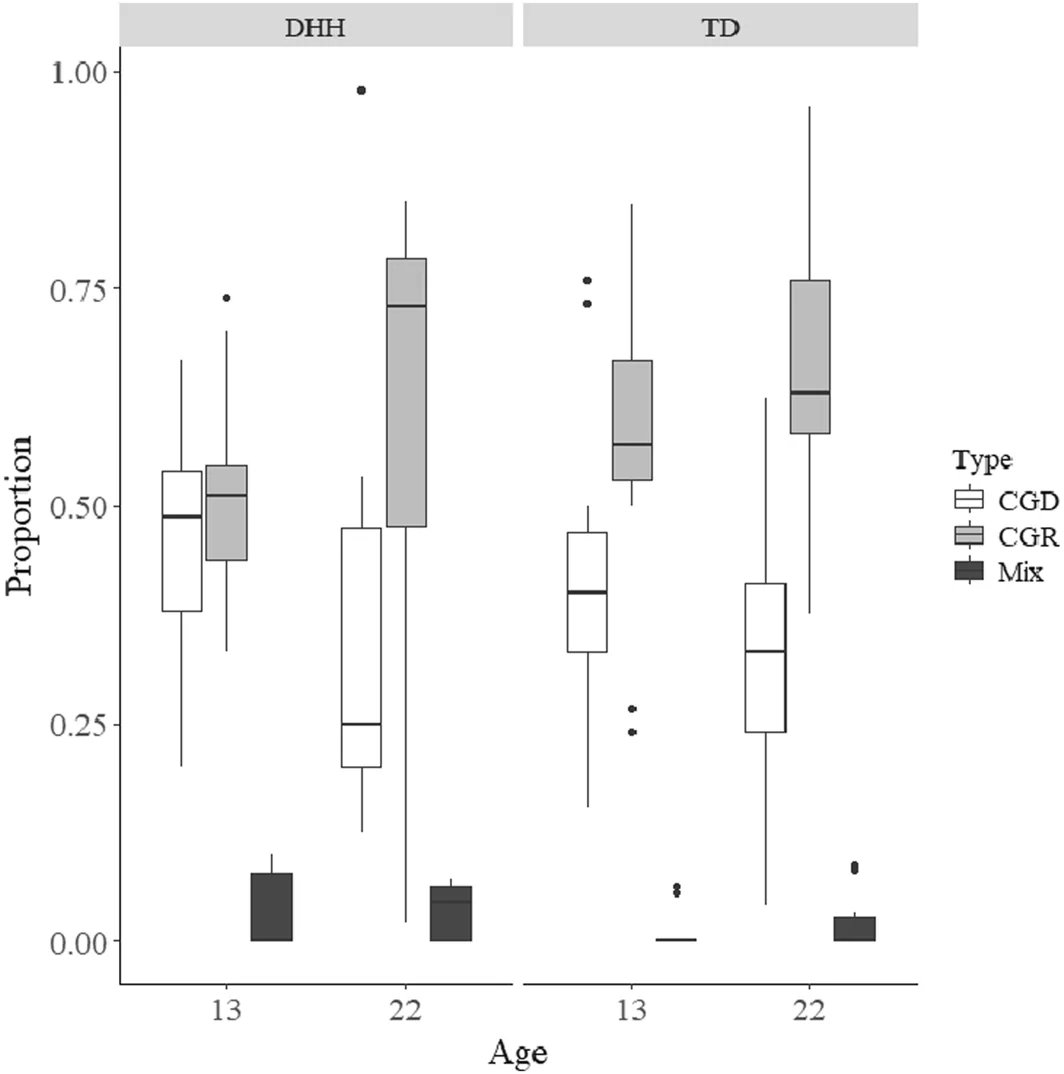
Proportion of multimodal verbs accompanied by depictive (CGD), relevant (CGR), or mixed caregiver actions in the input to DHH and TD infants at 13 and 22 months.

Next, we examined the types of actions that caregivers produced in proximity to their verb labels. With proportion of multimodal verbs accompanied by the different caregiver actions (CGD, CGR, mix) as our dependent variable, we fitted a linear mixed effects model with Age (13, 22), Type (CGD, CGR, Mix; CGD was the reference level), and their interaction as fixed effects, and by‐subject random intercepts (syntax: Proportion ∼ Age*Type + (1|Subject)). Results revealed no significant effect of age (*β* = −0.10, SE = 0.07, *t* = −1.58, *p* = 0.119), or the interaction between age and mix (*β* = 0.11, SE = 0.09, *t* = 1.13, *p* = 0.260) on the proportion measure. However, we found that the proportion of verbs accompanied by a CGR action was higher (*M* = 0.56, range = 0.02–0.85, SD = 0.19, 95% CI [0.48, 0.64]) than the proportion of verbs accompanied by a CGD action (*M* = 0.40, range = 0.13–0.98, SD = 0.20, 95% CI [0.31, 0.48]; *β* = 0.16, SE = 0.05, *t* = 3.46, *p* < 0.001; effect size estimate: 0.16, 95% CI [0.05, 0.27]), but the proportion of verbs accompanied by a Mix of actions (CGD and CGR) was lower (*M* = 0.03, range = 0.00–0.10, SD = 0.04, 95% CI [0.02, 0.05]) than that of verbs accompanied by a CGD action (*β* = −0.36; SE = 0.05; *t* = −7.94; *p* < 0.001; effect size estimate: −0.36, 95% CI [−0.48, −0.25]). Last, we also found a significant interaction effect between age and CGR (*β* = 0.21, SE = 0.09, *t* = 2.22, *p* = 0.03; effect size estimate: 0.21), such that this type of caregiver action, in comparison with CGD, was more common in the input to 22‐month‐olds (*M* = 0.61, range = 0.02–0.85, SD = 0.24, 95% CI [0.46, 0.76]) than 13‐month‐olds (*M* = 0.51, range = 0.33–0.74, SD = 0.12, 95% CI [0.43, 0.58]); see Figure 5.

**FIGURE 6:**
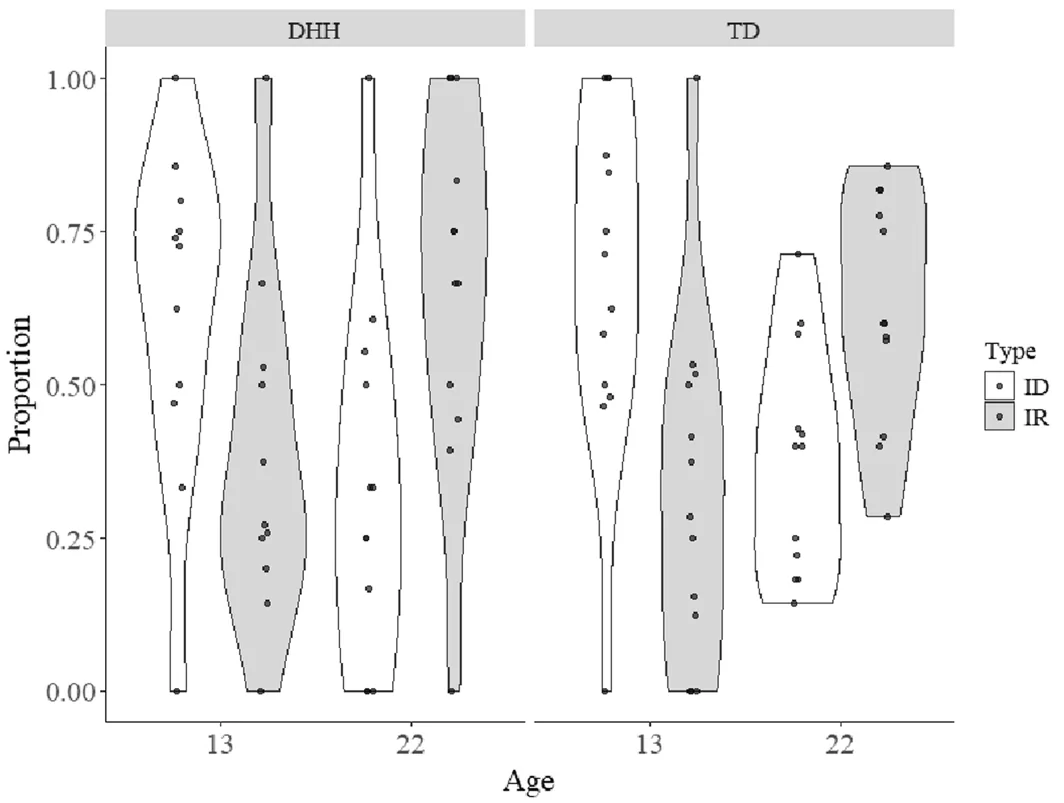
Proportion of multimodal verbs preceded by infant‐depictive (ID) or infant‐related (IR) actions in the input to DHH and TD infants at 13 and 22 months.

Last, we examined the types of infant actions that preceded their mothers' verb labels. With proportion of multimodal verbs accompanied by the different infant actions (ID, IR) as our dependent variable, we fitted a linear mixed effects model with Age (13, 22), Type (ID, IR), and their interaction as fixed effects, and by‐subject random intercepts (syntax: Proportion ∼ Age*Type + (1|Subject)). Results revealed a significant effect of age (*β* = −0.28, SE = 0.12, *t* = −2.37, *p* = 0.022) and the interaction between age and type (*β* = 0.57, SE = 0.17, *t* = 3.35, *p* = 0.001; effect size estimate: 0.57) on the proportion measure, such that out of the verbs that were preceded by an infant action, a higher proportion of them were ID at 13 months, whereas at 22 month, the majority of these events included an IR action. We found no significant effect of type (*β* = 0.05, SE = 0.09, *t* = 0.57, *p* = 0.572). This suggests that mothers of DHH infants change the way they use verbs to label infant actions; at 13 months, they use verbs that depict the actions their infants performed, whereas at 22 months, they use verbs that relate to the actions the infants performed but do not directly depict those actions; see Figure 6.

### 3.5 Multimodal Verb Labeling in the Input to TD Infants

With proportions of multimodal verbs per agent type as our dependent variable, we fitted a linear mixed effects model with Age (13, 22), Agent (caregiver, infant, co‐constructed; reference level was caregiver), and their interaction as fixed effects, and by‐subject random intercepts (syntax: Proportion ∼ Age*Agent + (1|Subject)). Results revealed no significant effect of age (*β* = 0.07, SE = 0.07, *t* = 0.99, *p* = 0.324), or the interaction between age and co‐constructed (*β* = −0.01, SE = 0.10, *t* = −0.11, *p* = 0.915) on the proportion measure. However, the interaction between age and infant was significant (*β* = −0.19, SE = 0.10, *t* = −2.00, *p* = 0.049; effect size estimate: −0.19); such that the proportion of verb labels preceded by an infant action compared to those followed by a caregiver action were more common in the input to 13‐month‐olds (*M* = 0.42, range = 0.13–0.75, SD = 0.20, 95% CI [0.29, 0.54]) as opposed to 22‐month‐olds (*M* = 0.29, range = 0.00–0.62, SD = 0.18, 95% CI [0.18, 0.40]).

We also found a statistically significant effect of agent, such that the proportion of verbs produced with caregiver action differed from those preceded by an infant action (*β* = −0.18, SE = 0.05, *t* = −3.73, *p* < 0.001; effect size estimate: −0.18, 95% CI [−0.3, −0.06]) and those that were co‐constructed (*β* = −0.43, SE = 0.05, *t* = −8.82, *p* < 0.001; effect size estimate: −0.43, 95% CI [−0.54, −0.31]); specifically, the proportion of verbs produced with caregiver action averaged 0.54 (range = 0.15–1.00, SD = 0.21, 95% CI [0.45, 0.62]), whereas those preceded by infant action, and verbs that were co‐constructed averaged 0.36 and 0.11 respectively (infant: range = 0.00–0.75, SD = 0.20, 95% CI [0.27, 0.43]; co‐constructed: range = 0.00–0.35, SD = 0.10, 95% CI [0.07, 0.15]). Further, verbs that were co‐constructed were significantly less frequent than verbs preceded by an infant action (*β* = −0.24, SE = 0.05, *t* = −5.09, *p* < 0.001; effect size estimate: −0.24, 95% CI [−0.36, −0.13]); see Figure 4. Thus, in the input to TD infants, verbs were more often accompanied by caregiver action rather than following infants' action or co‐constructed by the caregiver and the infant. At the same time, verbs following infant actions were more frequent in the input to younger infants.

Next, we examined whether the proportions of verb labels accompanied by each type of caregiver action (CGD, CGR, Mix) varied by age and type; we fitted a linear mixed effects model with Age (13, 22), Type (CGD, CGR, Mix; CGD was the reference level), and their interaction as fixed effects, and by‐subject random intercepts (syntax: Proportion ∼ Age*Type + (1|Subject)). Results revealed no significant effect of age (*β* = −0.10, SE = 0.05, *t* = −1.80, *p* = 0.075), or the interaction between age and mix (*β* = 0.10, SE = 0.08, *t* = 1.35, *p* = 0.18). However, we found that the proportion of verbs accompanied by a CGR action was higher (*M* = 0.61, range = 0.24–0.96, SD = 0.17, 95% CI [0.54, 0.67]) than the proportion of verbs accompanied by a CGD action (*M* = 0.37, range = 0.04–0.75, SD = 0.17, 95% CI [0.30, 0.44]; *β* = 0.24, SE = 0.04, *t* = 6.15, *p* < 0.001; effect size estimate: 0.235, 95% CI [0.14, 0.32]), but the proportion of verbs accompanied by a mix of actions (CGD and CGR) was lower (*M* = 0.02, range = 0.00–0.08, SD = 0.03, 95% CI [0.01, 0.03]) than that of verbs accompanied by a CGD action (*β* = −0.36, SE = 0.04, *t* = −9.36, *p* < 0.001; effect size estimate: −0.36, 95% CI [−0.45, −0.26]). We also found a significant interaction effect between age and CGR (*β* = 0.19, SE = 0.08, *t* = 2.48, *p* = 0.015; effect size estimate: 0.19), such that verbs related to caregiver's own actions (CGR), in comparison with those with CGD, were more common in the input to 22 month olds (*M* = 0.65, range = 0.38–0.96, SD = 0.17, 95% CI [0.55, 0.75]) than 13 month olds (*M* = 0.56, range = 0.24–0.84, SD = 0.16, 95% CI [0.46, 0.66]); see Figure 5.

Last, we examined the proportions of the types of infant actions that preceded mothers' verbs (ID, IR). We fitted a linear mixed effects model with Age (13, 22), Type (ID, IR), and their interaction as fixed effects, and by‐subject random intercepts (model syntax: Proportion ∼ Age*Type + (1|Subject)). We found a significant effect of age (*β* = −0.30, SE = 0.10, *t* = −3.12, *p* = 0.003) and the interaction between age and type (*β* = 0.61, SE = 0.14, *t* = 4.41, *p* < 0.001; effect size estimate: 0.61), such that out of the verbs that were preceded by an infant action, a higher proportion of them were depictive (ID) at 13 months, whereas at 22 months, more verbs were related to infant actions (IR). We found no significant effect of type (*β* = −0.06, SE = 0.07, *t* = −0.84, *p* = 0.408); see Figure 6. Here too, mothers of TD infants change the way they use verbs to label infant actions; at 13 months, they use verbs that depict the actions their infants performed earlier, whereas at 22 months, they use verbs that relate to the actions the infants performed but do not directly depict those actions.

## 4 Discussion

The current study provided the first detailed investigation of maternal multimodal verb input, and how it changes with infant development and is adapted to infants' hearing status in North American English‐speaking families. Using the Ambrose corpus (2016), we analysed 6 minutes of mother‐infant play interactions at 13 and 22 months in a sample of TD and DHH infants. For each verb the mother produced, we coded its type (action, mental) and its multimodal context: whether it was accompanied by a caregiver action, preceded by an infant action, or both (co‐constructed).

Consistent with our predictions and research indicating that caregiver speech becomes richer and more varied with age (Rowe 2012; West et al. 2023), mothers in both groups produced more verbs overall and more unique verbs at 22 months than at 13 months. However, the number of unique verbs was significantly higher in the input to TD infants than to DHH infants, suggesting that maternal verb input to DHH infants was less lexically varied overall. This finding aligns with previous work showing that hearing parents of DHH children use less complex language in their input, compared with parents who do not have a hearing mismatch with their children (Ambrose et al. 2015; Moeller and Schick 2006; Morgan et al. 2014; Spencer et al. 1992). At 13 months, DHH children in the present sample (*n* = 12) were among the larger group of DHH children (*n* = 25) who were reported to understand and produce fewer words from the Words and Gestures form of the MB‐CDI (Fenson et al. 2006) than their TD peers, and to use fewer spoken words during the recorded interactions, despite comparable gesture use (Ambrose 2016). Using the original MB‐CDI administrations, we found that these patterns hold in our current sample, which is half of the original sample in Ambrose (2016), both for production (Wilcoxon two‐sample test: *Z* = −2.004, *p* = 0.022) and comprehension (Wilcoxon two‐sample test: *Z* = −2.697, *p* = 0.003). Therefore, the reduced lexical diversity we observed in verb input may reflect mothers' attempts to adjust their input to their children's spoken language skills. However, a more crucial investigation would have been to explore the item‐by‐item MB‐CDI data to specifically examine whether maternal verb input at 13 months was related to toddlers' verb comprehension at that time point; unfortunately, we could not complete these analyses as we did not have access to the full MB‐CDI administrations. Further, given that the MB‐CDI was not administered at 22 months, we do not know whether verb input diversity tracked children's lexical growth over time. Untangling both of these links requires investigating the relationship between specific features in the input of hearing parents and their DHH infants' language skills, and examining parents' own views about the patterns observed in their input (e.g., are they actively simplifying the verb input?).

Unpacking this verb input in more detail revealed that mothers opted for using verbs whose meaning is less abstract: action verbs made up almost 60% of verb input to infants regardless of age or hearing status. Even as verb input grew in frequency and variety, mothers continued to rely mostly on verbs with concrete, observable referents that are closely linked to infants' embodied experiences (Golinkoff and Hirsh‐Pasek 2008). This pattern possibly reflects the nature of the interactions we studied: play interactions invite talk about ongoing actions and activities. In their study of language input to 13‐month‐olds, Tamis‐LeMonda et al. (2019), for example, found that 72% of maternal utterances during play at home were referential, that is, provided information about objects or activities. Although referential talk includes mental verbs too, play interactions offer many more opportunities to name actions than mental states. Mothers in our study knew they were being videotaped and were playing with their infants in an unfamiliar room. Both factors may have led dyads toward object‐focused play, where talk centers on the here‐and‐now and thus action verbs come up more than mental verbs. How verb types are distributed across settings and activities, particularly in symbolic play where talk becomes more decontextualized, is a question for future studies.

With respect to multimodally packaged verbs, our findings confirm the previously reported verb‐action co‐occurrence (Nomikou et al. 2017; Rodrigo et al. 2020): most caregivers' verbs were tightly linked with actions in the input to their DHH and TD infants at 13 and 22 months. Yet, contrary to our prediction, but in line with previous work (Rodrigo et al. 2020; West et al. 2022), the proportion of multimodal verbs did not decrease with age, suggesting that caregivers keep drawing on multimodal cues for verb labeling throughout the second year, a period of rapid lexical growth, particularly for verb acquisition. However, it is possible that specifically for multimodal verbs, a developmental decline occurs later in development. Here, the scarce documentation of the developmental trajectory of multimodal input makes it difficult to interpret this finding. On the one hand, theoretical (Gogate et al. 2001) and experimental accounts (Gogate et al. 2000; Ko et al. 2023) suggest a decrease in multimodal input across time. Yet, experimental studies are based on small samples and do not discriminate between nouns and verbs or focus on nouns only. On the other hand, research on the trajectory of verb learning suggests that children's verb production increases in the second year (Fenson et al. 2006). Thus, given that research on child directed behavior in general suggests that it changes along with the child's development, it is reasonable to assume that even at 22 months of age, mothers still provide multimodal verbs because their children are *still* developing their verb vocabulary.

Further, in line with our predictions, mothers of DHH infants produced a higher proportion of multimodal verbs compared to mothers of TD infants. This is not only consistent with previous research comparing parents of hearing children and those of DHH children (Depowski et al. 2015) but also highlights the adaptive nature of language environments (Gogate et al. 2001). Specifically, parents of DHH infants tune in to their children's reduced/degraded access to the auditory signal which might be variable across everyday contexts leading to situations where linguistic information is even more fleeting than usual and is missed. As such, parents utilize other sensory modalities more frequently to reinforce or supplement the speech signal thus making up for the missed information. Learning verbs in particular requires children to pay attention to the timing and sequence of actions—all of which can be emphasized multimodally (Vigliocco et al. 2019); however, if such information—in part or in full—is missed through lack of access to the full range of the auditory signal, then increasing the use of multimodal packaging of verbs can be an excellent scaffolding strategy. And while we do not know whether such strategies help DHH infants learn verbs, some studies indicate that multimodally scaffolded nouns can be learned better than those that are not multimodally scaffolded (Gogate et al. 2006; Seidl et al. 2024; Tincoff et al. 2018); future work can extend these findings to verb learning to shed light on the implications of the increased use of multimodal verbs with DHH infants.

Zooming in on multimodal verbs, we examined whether mothers' verbs matched their actions, their infant's actions, or were co‐constructed. Based on studies showing that infants' actions shape the input they receive (Suarez‐Rivera et al. 2022; Yu and Smith 2012), we expected mothers to produce verbs in response to infants' actions. However, and in line with Rodrigo et al. (2020), across groups, caregivers produced a higher proportion of verbs that were related to their own actions than to infant‐actions or co‐constructed episodes. We also expected that as infants become more active, parents will follow their actions more, and thus, verbs matching infant actions will increase over time. Yet, we found no effect of age on the proportion of these infant‐led verb labels; but we did find different developmental paths for TD and DHH infants. Verbs that were produced in response to infant actions were more common in the input to 13‐month‐olds than 22‐month‐olds who were TD, suggesting that earlier in development, caregivers provide input that is more temporally linked to the infant's actions and interest. In DHH dyads, the proportion of infant‐led multimodal verbs did not change with development, suggesting that caregivers maintain similar levels of responsiveness to infants' actions.

Last, we also examined verb‐action co‐occurrences to determine whether the accompanying action depicted the meaning of the verb—either through caregivers' own actions or through verbs describing infants' actions—or whether the verb and action were only related. Across both groups, when mothers produced verbs accompanied by their own actions, the actions were more often related to the verb's meaning than directly depictive. This pattern was observed at both ages but was more pronounced at 22 months. As infants grew older, caregivers increasingly accompanied verbs with actions that situated the verb within a broader activity or event, rather than demonstrating the meaning of the verb. This pattern fits with early findings suggesting that caregivers' gestures are not predominantly redundant or depictive, but instead often complement speech by providing additional information rather than simply reinforcing it (Iverson et al. 1999). A different and clearer developmental pattern emerged when examining verbs produced in response to infants' actions. At 13 months, mothers typically produced verbs that named what the infant was doing, closely linking the verb to the infant's ongoing, observable action and sensorimotor experience. By contrast, at 22 months, mothers more often produced verbs that were related to infant actions without directly naming them, for example by referring to outcomes or associated activities rather than to the visible movement itself. These findings suggest that early in development, caregivers provide verb input that closely links words to immediately observable actions, offering a transparent basis for early verb learning (Perniss and Vigliocco 2014). Later in development, caregivers increasingly connect verbs to broader aspects of events rather than providing labels for actions. This pattern fits with the idea that children rely on different sources of information when learning verbs at different ages (Golinkoff and Hirsh‐Pasek 2008; Arunachalam et al. 2013). Early verb learning is thought to rely heavily on perceptually salient, here‐and‐now information, with increasing use of social‐pragmatic and linguistic cues as children's representational capacities develop (Hollich et al. 2000). This shift in multimodal packaging may reflect an adaptation of caregiver input to children's growing ability to learn verbs from information that is not linked to one's own experience or is depictive of the meaning of words.

### 4.1 Limitations and Future Directions

Our study used a fine‐grained descriptive approach to investigate multimodal verb input in a longitudinal and comparative design, a hitherto underexplored perspective of early language development. The detailed coding of verb types, referents of multimodal input, and the specific form of multimodal verb input in terms of its depictive qualities allowed us to generate rich observations of the ways in which verb learning is scaffolded within play interactions and how it changes throughout development and in the input to TD and DHH infants.

Yet, the opportunities provided by any approach always come with constraints and limits. In this sense, the detailed coding used for the present analyses enabled us to focus on a relatively brief window of free play interaction, a context that might strongly support dense multimodal coordination. While this is an established method in the field, it may limit the generalizability of our findings to everyday contexts and mundane less‐focussed interactions. Future studies across longer time windows and more varied interactional contexts may be more sensitive to developmental and contextual differences. Additionally, the DHH group was treated as a single category despite known heterogeneity in auditory access and device use. Children in the current sample were implanted at different time points, and as such their hearing experiences varied. Such variability may shape how caregivers adapt their language input in interaction. While our small sample did not allow for further analyses, future studies with larger samples could model these differences more explicitly. Further, to properly unpack the impact of hearing experiences on multimodal input, DHH children will need to be compared not only to their chronological age matches, but also their hearing age matches; such comparisons will allow us to investigate how various durations of hearing experiences impact multimodal input beyond age alone. Furthermore, additional measures of children's own language abilities were also limited with CDI data available only at 13 months and no repeated or standardised measures across time. We also did not have an item‐by‐item breakdown of the CDI data, which would have allowed us to explore the specific verbs that children understood and used and to relate these with the mothers' multimodal verb scaffolding, clarifying how multimodal verb input relates to individual differences in children's verb acquisition. Finally, our coding did not systematically distinguish between gestures and sign language. Given evidence that signing caregivers also tend to produce a wider range of gestures (Ambrose 2016), some manual behaviors in the DHH group may reflect overlapping gestural and signed communication. Future work using annotation schemes that explicitly differentiate gestures from sign language would be needed to disentangle their respective contributions to multimodal verb input. Last, we must also acknowledge that our small sample—which is not uncommon in studies with DHH infants—may have limited our ability to detect smaller effects.

## 5 Conclusions

In sum, our analyses revealed that verb input to TD and DHH infants is multimodally packaged and constructed in comparable ways, albeit with some notable differences. DHH infants heard less diverse verb input yet received a higher proportion of multimodally packaged verbs. These two features seem to go hand in hand: mothers of DHH infants did not overload the play interactions with lexically diverse verbs, and more frequently supported the verbs they did produce with multimodal cues. Such behaviors might reflect advice that these mothers had received in speech therapy sessions or might stem from their own interactional experience with their child and the calibration toward specific communication strategies. Importantly, the current study highlights the relevance of multimodal input throughout development and is a first important step in unpacking the multimodal nature of language learning in hearing and DHH infants.

## Author Contributions

**Feile Meng:** data curation (annotating, managing data, equal), writing – original draft (lead). **Isobel Horsfall‐Turner:** data curation (annotating, managing data, equal). **Cassim Hussain:** data curation (annotating, managing data, equal). **Iris Nomikou:** conceptualization (equal), project administration (equal), supervision (equal), writing – review and editing (co‐lead). **Rana Abu‐Zhaya:** conceptualization (equal), project administration (equal), supervision (equal), formal analysis (lead), writing – review and editing (co‐lead).

## Funding

The authors have nothing to report.

## Ethics Statement

The study was conducted in accordance with ethical standards set forth by University College London and the University of Portsmouth, as well as the guidelines set forth by the TalkBank community for use of available corpora in the CHILDES database.

## Conflicts of Interest

The authors declare no conflicts of interest.

## Supporting information


Supporting Information S1

## Data Availability

All videos and transcripts used in the current study are accessible from the CHILDES database (MacWhinney 1995); raw data generated based on the coding of these videos as well as the coding template and analysis code are available upon request.
