# Supplementary material for: Unpacking Multimodal Verb Input in English: Insights From Typically Developing Infants and Those Who are Deaf and Hard of Hearing
Source: Infancy. 2026 Sep 20;31(5):e70127. doi: 10.1111/infa.70127 (PMC13590040; doi:10.1111/infa.70127)
Supplement: Supplementary file 1 [file INFA-31-0-s001.docx]

**Supplementary Materials**

## **Data Coding**

**Coding Team**

Videos were coded using ELAN (Version 6.7; Wittenburg et al., 2006) by three research assistants (RAs) who were based in two different research labs and were supervised by the last two authors (respectively, PI1 & PI2). The coding process followed the guidelines set forth in the coding manual (see Table 1) collaboratively established by the research teams in the two research labs. Coders were asked to annotate the verbs that mothers produced, followed by marking the 3-seconds preceding and following each verb, indicating the labelling episode. They were then asked to watch the labelling episode for each verb and examine whether it included a caregiver or infant action, or both. Once an action was identified, it was coded in line with the coding categories described in Table 1. Thus, coders were tasked with coding each behaviour (verb, caregiver action, infant action) separately, rather than using their insights from watching the videos to classify the verbs as multimodally reinforced or not.

The first batch of coding (*n* = 20) was completed by an RA who was trained by PI1. This RA initially coded a file and discussed all annotations with PI1. Then they independently coded all the files. Regular meetings with PI1 were used to discuss problematic cases and agree on coding. The remaining files (*n* = 30) were coded by the other two RAs who were based in the second research lab and were trained by the last author, PI2. Initially, these two coders collaboratively coded two files, which were then submitted to PI2 for review. Following on from this, coding was completed independently by each of the two coders, each handling a comparable amount of data.

**Table 1**
*Coding Manual*

| Name | ELAN Code | Definition | Examples |
| --- | --- | --- | --- |
| Caregiver- depictive | CGD | Caregiver models the action described by the verb, there is a clear performance of the action referred to by the verb.  Includes the caregiver physically manipulating the infant into performing the action. | - Caregiver states “let’s *put* the doll in the crib” whilst putting the doll into the crib.  - Caregiver states “*jump*” whilst lifting the infant up and down |
| Caregiver- related | CGR | Any verb-relevant action performed by caregiver which does not directly model the action described by the verb.  Includes caregiver pointing unless accompanied by the verb *point.*  Includes object manipulation which is relevant to the verb.  Does not include pointing which is irrelevant to the verb (e.g. *say*).  Does not include general object manipulation (e.g. *look* at this whilst picking something up). | - Caregiver states “*look* here” whilst pointing at a picture  - Caregiver states “*bring* that over here” whilst beckoning  - Caregiver states “*feed* the baby” whilst offering utensils |
| Infant- depictive | ID | The infant performs an action which the caregiver labels with the associated verb. Includes cases where infant begins action trajectory prior to verb labelling but completes the action post-verb. Does not include cases where the infant initiates the verb-relevant action after the verb. | - Infant shakes a toy and caregiver states “*shake*”  - Infant sits down and caregiver states “you can *sit* there”  - Infant gurgles and caregiver states “can you *tell* me some more?” |
| Infant- related | IR | The infant performs an action and the caregiver utters a verb which is relevant but not directly describing the infant action. | - Infant picks up a book and caregiver states “shall we *read* some of that?” |
| Not codable | NC | Action cannot be coded due to no clear view of subject(s) |  |

## **Data Checking and Inter-rater Reliability**

Data coding was rigorously checked in various ways to ensure accuracy and inter-rater reliability. First, a total 14 files (including the two jointly coded by the two RAs) underwent a detailed review by the last author (PI2) who checked if coding was consistent with the manual guidelines. The review was conducted on batches of 2-3 files that were coded jointly/separately by the two RAs who were in the same lab. After the review, detailed feedback was provided to the RAs, who then made necessary adjustments to the coded files. Second, one of the two RAs checked all 20 videos that were previously coded by the RA in the other research lab and fixed any discrepancies they detected. Third, each of the two RAs cross-checked 8-9 randomly selected files that were coded by the other RA. This process involved meticulously checking the file to identify any verbs that had been missed and to ensure verb boundaries were marked precisely, covering the whole verb while excluding the sounds of preceding or following words. Moreover, both coders checked for any caregiver or infant actions near the verbs that might have been left unmarked and confirmed that the actions coded were categorised correctly (e.g., CGD/CGR or ID/IR). Of these files that were checked and fixed by the RAs who were in the same lab, we randomly selected 6 (12% of the sample) for inter-rater reliability. Here, using the Intraclass correlation coefficient (ICC), we examined the levels of agreement in the number of events (verbs, CGD, CGR, ID, IR) that each of the coders had in their final version of each of the 6 files. Our analyses showed that the coders had high levels of agreement for all types of events, as seen in Table 2 below.

These thorough checking procedures, along with constant communication between the RAs and the PIs at both research labs, significantly minimized errors and enhanced the overall quality of the data.

**Table 2**

*Inter-rater Reliability (ICC) by Event Type*

| Type of event | ICC [95% CI] |
| --- | --- |
| Verbs | 1.000 [0.999, 1.000] |
| CGD | 0.989 [0.811, 0.999] |
| CGR | 0.897 [0.413, 0.985] |
| ID | 0.989 [0.926, 0.999] |
| IR | 0.923 [0.558, 0.989] |
